# EMPOWERING ALL AGES THROUGH INTERGENERATIONAL CONNECTION AND LIFELONG LEARNING

**DOI:** 10.1093/geroni/igad104.0082

**Published:** 2023-12-21

**Authors:** Lisa Hollis-Sawyer, Laura Donorfio

## Abstract

This symposium will share a variety of pedagogical approaches designed to foster age-inclusivity and intergenerational learning. The authors will share research and outcomes of different educational designs utilizing intergenerational programming and other approaches to engage older and younger adult learners. The first presentation will explore how intergenerational discussions can be used in the classroom to facilitate collaborative learning among older adults, students, and instructors. The second presentation will discuss the development of a course abroad in Scotland called “Global Aging & Age-Friendly Initiatives,” aiming to promote intergenerational connection and global age-inclusivity. The third presentation will discuss “Between Islands,” an intergenerational virtual contact intervention designed to promote creativity and intergenerational connection between younger and older adults while challenging stereotypes about aging. The fourth presentation will explore the experiences of emeriti faculty in engaging online with Introduction to Gerontology students in a novel intergenerational classroom activity. The final presentation will review intergenerational classroom design guidelines to optimize multiple generations’ learning experiences, aging understanding/appreciation of others, and age-inclusivity. The empowerment and benefits of these efforts for the life-long learning of the generations involved will be discussed.

